# High-Level Secretory Expression of Recombinant Type XVII Human-like Collagen in *Komagataella phaffii*

**DOI:** 10.3390/ijms27125613

**Published:** 2026-06-22

**Authors:** Yixuan Jia, Junhao Yue, Wanting Wu, Weirui Zhao, Sheng Hu, Lehe Mei, Peilian Wei, Changjiang Lyu

**Affiliations:** 1 School of Biological and Chemical Engineering, Zhejiang University of Science and Technology, Hangzhou 310023, China; 17745008004@163.com (Y.J.); wt152612@sina.com (W.W.); 2 School of Biological and Chemical Engineering, Ningbo Tech University, Ningbo 315100, China; zhaowrzju@zju.edu.cn (W.Z.); genegun@zju.edu.cn (S.H.); meilh@zju.edu.cn (L.M.)

**Keywords:** type XVII collagen, *Komagataella phaffii*, secretory expression, human foreskin fibroblasts, immortalized keratinocytes

## Abstract

Type XVII collagen (COL17) is crucial for skin integrity but difficult to produce. To achieve high-level secretory expression, a human COL17 segment was designed and cloned into the pPIC9K vector with six different α-mating factor signal peptides and integrated into *Komagataella phaffii* GS115. Fed-batch fermentation in a 5 L bioreactor yielded 4.7 g/L of recombinant COL17. Functional assays showed that it promoted the proliferation of human skin fibroblast (HFF) and immortalized keratinocytes (HaCaT), upregulated *COL1A1*, *COL3A1*, and *TIMP1* in HFF cells, and enhanced skin barrier-related genes (*KRT1*, *KRT5*, *KRT10*, *KRT14*, *IVL*, *LOR*, *FLG*) in HaCaT cells. In a UVB-induced photoaging model, COL17 reduced reactive oxygen species (ROS) and matrix metalloproteinase 3 (MMP3) activity. This recombinant collagen exhibits photoprotective, regenerative, and barrier-enhancing activities, offering potential for skincare and tissue engineering.

## 1. Introduction

Collagen is a natural biopolymer that serves as the fundamental structural and functional framework of human connective tissues, accounting for nearly one-third of the total protein in the body [1]. It plays a central role in maintaining the structure of the extracellular matrix (ECM), mediating cell–cell interactions, and coordinating tissue regeneration [2]. Due to their excellent biocompatibility, collagen-based biomaterials are widely applied in the repair of bone, cartilage, and skin defects, as well as in dental implants [3]. Furthermore, human collagen types I, II, III, V, X, and XVII serve as key components in a range of consumer products, including skincare formulations, nutritional supplements, foods, and beverages [4].

Type XVII collagen (COL17), a transmembrane protein localized in the epidermal basement membrane, regulates the differentiation of hair follicle stem cells and plays an essential role in anchoring both hair follicle and pigment stem cells to the basement membrane [5]. Unlike fibrillar collagens that only support ECM structures [6], this anchoring mediated by COL17 is critical for maintaining the self-renewal capacity and differentiation potential of these stem cells, as well as for ensuring the proper differentiation and supply of hair matrix cells derived from them [7,8]. Strict regulation of COL17 is crucial, as its decreased expression is associated with a range of aging-related skin phenotypes, including atrophy, increased fragility, discoloration, and alopecia [9,10].

As COL17 is a transmembrane protein, direct administration from outside the living body is thus challenging [11]. Additionally, expressing full-length COL17 in microorganisms is difficult, necessitating the selection of functional fragments for microbial biosynthesis. In previous studies, several COL17 fragments were heterologously expressed and purified in both *Escherichia coli* and *K. phaffii* systems [12]. The recombinant COL17 protein constitutes a promising and competitive biomaterial that mirrors the skin repair and regenerative properties of its natural counterpart, effectively stimulating cell proliferation and mitigating damage from UV radiation [13,14]. However, fed-batch fermentation of the recombinant strains yielded less than 690 mg/L [15]. Obviously, the efficient expression of COL17 is therefore pivotal for progressing functional studies and broadening its applications.

To overcome this limitation, a COL17 fragment enriched with highly hydrophilic domains was designed based on the core sequence of human COL17A1. Subsequently, the efficient secretion of the engineered protein was achieved in the *K. phaffii* host, accomplished through strategic signal peptide selection and multi-copy genomic integration. Furthermore, its effects on proliferation and key gene expression in human foreskin fibroblasts (HFFs) and immortalized keratinocytes (HaCaTs) were assessed to elucidate the molecular mechanisms underlying its skin repair activity and its ability to alleviate skin photoaging induced by ultraviolet radiation. Overall, this study aims to explore the potential of recombinant COL17 as a protector of the skin basement membrane, with promising implications for future medical and cosmetic applications.

## 2. Results and Discussion

### 2.1. Effects of Signal Peptide on Heterologous COL17 Expression in K. phaffii

To ensure high solubility and proper folding while maintaining biological function, the recombinant COL17 sequence was designed based on a systematic screening of hydrophilic regions within the extracellular domain of the human COL17 α1 chain. As detailed in Table S1, eleven candidate regions spanning residues 575–1482 were evaluated using the Grand Average of Hydropathy (GRAVY) index. The optimal sequence was selected based on its favorable hydrophilicity and the inclusion of functional domains critical for cell adhesion [16]. Since the secretion efficiency of heterologous proteins in *K. phaffii* is highly dependent on the compatibility between the signal peptide (SP) and the target protein [17], five α-mating factor (α-MF) signal peptides derived from different yeast species were evaluated. Among these, the sequences from *Wickerhamomyces ciferrii* and *Saccharomyces cerevisiae* represent two prominent variants [18,19]. Given that α-MF signals mediate co-translational translocation [19], structural differences in their pro-peptide regions may critically affect their interaction with the secretory machinery. Therefore, empirical screening of these diverse α-MF origins provides a rational strategy for identifying the optimal signal peptide for maximizing COL17 secretion [17,19].

**Figure 1:**
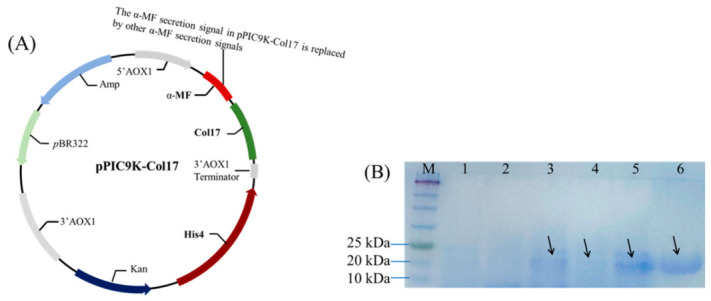
(**A**) Schematic of the recombinant expression vector. (**B**) SDS-PAGE analysis of fermentation supernatants from recombinant *K. phaffii* strains expressing COL17. Lane M: protein marker. Lanes 1–6: culture supernatants from independent transformants that were mediated by the α-MF secretion signals of *Geotrichum candidum*, *Lachancea dasiensis*, *Millerozyma farinosa*, *Saccharomyces cerevisiae*, *Tetrapisispora phaffii*, and *Wickerhamomyces ciferrii*. Lanes 3–6 exhibit a prominent band at ~20 kDa, which matches the predicted molecular weight of recombinant COL17.

The specific sequences of the signal peptides tested are listed in Table S3. The recombinant plasmids (Figure 1A) were generated by integrating the designed fusion genes into pPIC9K using Gibson assembly and further verified via DNA sequencing. After linearization with *Sac*I, the plasmids were transformed into competent *K. phaffii* GS115 cells. Transformants with multi-copy genomic integration were subsequently screened on YPD medium supplemented with 4 mg/mL G418. SDS-PAGE analysis revealed that the tested α-MF secretion signals from *Millerozyma farinosa*, *Saccharomyces cerevisiae*, *Tetrapisispora phaffii*, and *Wickerhamomyces ciferrii* successfully mediated the secretion of recombinant COL17 (Figure 1B). Notably, the secretion efficiency conferred by the α-MF secretion signal from *W. ciferrii* was higher than that from *S. cerevisiae*, indicating its potential to replace the *S. cerevisiae* α-MF signal for enhancing COL17 secretion in *K. phaffii*.

### 2.2. Purification of Recombinant COL17

**Figure 2:**
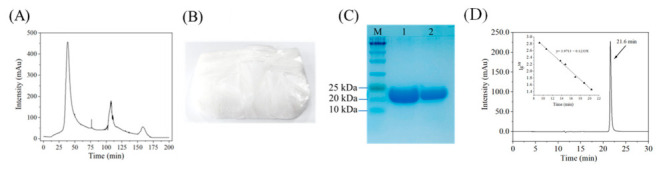
(**A**) Size-exclusion chromatography on Sephadex G-25 column, (**B**) freeze-dried fiber of recombinant COL17, (**C**) SDS-PAGE analysis of recombinant COL17, Lane M, protein marker; lanes 1 and 2, purified recombinant protein 17, (**D**) SEC-HPLC analysis of recombinant COL17.

The elution profile from the initial size-exclusion chromatography step showed a single major peak, indicating effective separation (Figure 2A). After lyophilization, the purified product appeared as a white powder (Figure 2B). SDS-PAGE analysis revealed a single distinct band at approximately 20 kDa with no visible impurities, consistent with the expected molecular weight (Figure 2C). The fermentation yield reached 4.7 g/L. The molecular weight distribution of the purified protein was analyzed via size-exclusion chromatography (SEC-HPLC) on a Xtimate SEC-300 column (300 mm × 7.8 mm, 5 μm). The chromatogram exhibited a single, symmetric peak with a retention time of approximately 21.6 min (Figure 2D). The molecular weight markers shown in the inset (a–g) are thyroglobulin (669 kDa), ferritin (440 kDa), myosin (200 kDa), aldolase (158 kDa), bovine serum albumin (67 kDa), ovalbumin (45 kDa), and bovine carbonic anhydrase (29 kDa). Based on the calibration curve generated using standard protein markers, the molecular weight was estimated to be 20.3 kDa, which is consistent with the theoretical size of the monomeric target protein (20.6 kDa). No significant peaks corresponding to high-molecular-weight aggregates or low-molecular-weight degradation fragments were detected. These results indicate that the recombinant collagen is highly pure and homogeneous, a quality attribute essential for reliable performance in subsequent bioactivity assays [20].

The heterologous production of COL17 has been explored in several host systems with diverse outcomes. In *E. coli*, Wang et al. utilized a TrxA fusion strategy for a 16-repeat COL17 fragment (27–33 aa motif), obtaining 223 mg/L in shake flasks and scaling up to 0.6 g/L in a 5 L fed-batch process [15]. In yeast, *P. pastoris* (*K. phaffii*) X-33 transformed with a pPICZαA plasmid encoding a six-tandem-repeat COL17 variant reached an impressive 10.32 g/L post-methanol induction (48 h) in a 5 L bioreactor [13]. In this work, the *K. phaffii* system, which employs AOX1-driven secretory expression and the *W. ciferrii* α-MF signal peptide, attained 4.7 g/L under fed-batch conditions. Such comparative data highlight that while *K. phaffii* systems can support robust COL17 biosynthesis, maximizing titers requires careful optimization of both genetic constructs and bioprocess parameters.

### 2.3. Effects of Recombinant COL17 on HaCaT and HFF Cell Proliferation

**Figure 3:**
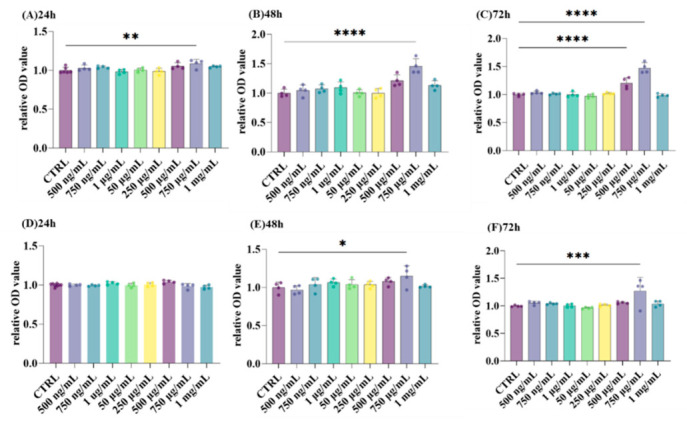
All panels show relative OD values normalized to the untreated control: (**A**–**C**) HaCaT cells at 24 h, 48 h, and 72 h; (**D**–**F**) HFFs at 24 h, 48 h, and 72 h. Cells were treated with various concentrations of COL17 (500 ng/mL to 1 mg/mL), and cell viability was assessed with a CCK-8 assay. Data are presented as mean ± SD (*n* = 3). Statistical significance versus control: * *p* < 0.05, ** *p* < 0.01, *** *p* < 0.001, **** *p* < 0.0001.

Cell proliferation is essential to skin repair and regeneration. To evaluate the proliferative activity of recombinant COL17 collagen, its effects on HaCaT and HFF cells were assessed using the CCK-8 assay [21]. As shown in Figure 3, recombinant COL17 did not induce cytotoxicity in either cell line at concentrations up to 1 mg/mL. Notably, the proliferative response exhibited a non-linear, bell-shaped trend. While lower concentrations showed modest effects, 750 μg/mL emerged as the optimal threshold, yielding the most significant promotion of fibroblast proliferation. Similarly, treatment with 750 μg/mL COL17 significantly enhanced keratinocyte proliferation at 48 and 72 h (*p* < 0.0001). Crucially, concentrations exceeding this optimum (e.g., 1 mg/mL) did not result in further enhancement, suggesting a saturation of integrin-binding sites or steric hindrance at high ligand densities [22,23]. This specific bioactivity is likely mediated by COL17-enhanced cell adhesion and extracellular matrix interactions [24]. Therefore, 750 μg/mL was selected as the optimal dose for subsequent experiments.

### 2.4. Effects of Recombinant COL17 on the Expression of Collagen and Skin Barrier-Related Protein Genes in HFF and HaCaT Cells

**Figure 4:**
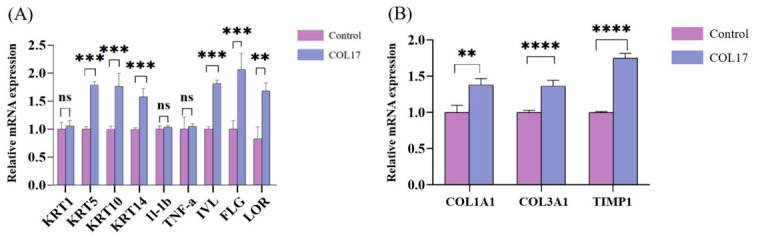
Effects of recombinant COL17 on the expression of extracellular matrix and skin barrier-related genes in human skin fibroblasts (HFFs) and immortalized keratinocytes (HaCaTs). (**A**) Effects of treatment with 750 μg/mL COL17 for 48 h on the expression of keratin and differentiation-related genes. (**B**) Effects of treatment with 750 μg/mL COL17 for 48 h on collagen-related gene expression. Cells were treated with 750 μg/mL COL17 for 48 h, and mRNA levels were analyzed via qPCR. Data are presented as mean ± SD (*n* = 3 independent experiments). All panels show relative mRNA expression normalized to GAPDH. Statistical significance versus control: ** *p* < 0.01, *** *p* < 0.001, **** *p* < 0.0001; ns, not significant (*p* > 0.05).

Previous studies have shown that COL17 plays a key role in promoting skin homeostasis via dual-tissue regulation [25]. To evaluate the effects of recombinant COL17 on epidermal barrier function, inflammatory response, and dermal extracellular matrix (ECM) synthesis, a panel of genes representing these key biological processes were selected. As shown in Figure 4A, following a 48 h treatment with 750 μg/mL of recombinant COL17, the mRNA expression of canonical markers for epidermal integrity, multiple keratin genes (*KRT1*, *KRT5*, *KRT10*, *KRT14*), and skin barrier protein genes (*IVL*, *LOR*, *FLG*) was significantly upregulated in the HaCaT cells, indicating enhanced keratinocyte differentiation and barrier formation at the transcriptional level. In contrast, no significant changes were observed in the expression of inflammation-related cytokine genes *IL1B* and *TNF* (*p* > 0.05, ns). The selective upregulation of barrier-related genes, without inducing inflammation, indicates that the recombinant COL17 promotes molecular barrier function and maintains immune quiescence. This supports its potential to enhance skin repair and foster a favorable microenvironment. Fibroblasts serve as the principal cellular entities responsible for the synthesis of the dermal extracellular matrix (ECM) [26]. As shown in Figure 4B, qPCR analysis revealed that treatment with 750 μg/mL of recombinant COL17 significantly upregulated the expression of major ECM component genes, including *COL1A1*, *COL3A1*, and the matrix metalloproteinase inhibitor *TIMP1*, in HFF cells (*p* < 0.05 to *p* < 0.0001 vs. control). Collectively, by concomitantly enhancing ECM synthesis in HFFs and upregulating skin barrier-related gene expression in HaCaT cells, recombinant COL17 acts in a manner consistent with the concept of dermal–epidermal crosstalk [4].

### 2.5. Protective Effect of Recombinant COL17 Against UV-Induced Dermal Damage

**Figure 5:**
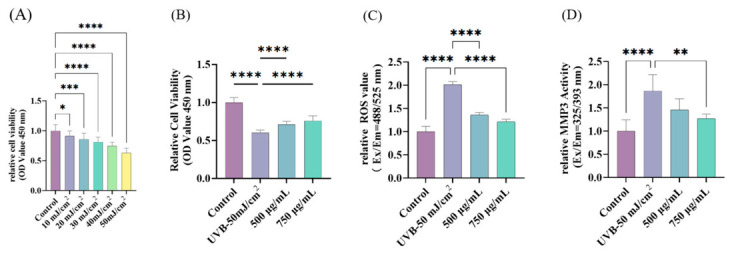
Protective effect of recombinant COL17 against UV-induced dermal fibroblast damage. (**A**) Effect of different doses of UVB irradiation on fibroblast viability. (**B**) Effect of different concentrations of recombinant COL17 on the viability of UVB-irradiated fibroblasts. (**C**) Effect of 50 mJ/cm^2^ UVB irradiation on ROS levels in fibroblasts treated with different concentrations of COL17. (**D**) Effect of 50 mJ/cm^2^ UVB irradiation on MMP3 activity in fibroblasts treated with different concentrations of COL17. Data are presented as mean ± SD (*n* = 3 independent experiments). Statistical significance versus control: * *p* < 0.05, ** *p* < 0.01, *** *p* < 0.001, **** *p* < 0.0001.

Previous studies have shown that a low-molecular-weight recombinant human COL17-like protein has high biocompatibility and maintains the skin repair functions of native COL17 [27]. The mechanism entails the suppression of MMP3 upregulation and collagen degradation through a reduction in oxidative stress, which in turn alleviates UV-induced damage. Thus, the protective effect of recombinant COL17 against UV-induced damage in keratinocytes and fibroblasts was also investigated in this work. Fibroblasts were cultured with 500 μg/mL or 750 μg/mL recombinant COL17 and subjected to UVB irradiation to establish a cellular injury model. The levels of reactive oxygen species (ROS) and matrix metalloproteinase MMP3 were then measured to assess the protective effect of recombinant COL17 against UVB-induced damage. As shown in Figure 5A, UVB irradiation at 10–50 mJ/cm^2^ decreased fibroblast viability in a dose-dependent manner; accordingly, 50 mJ/cm^2^ was employed in subsequent experiments. Figure 5B demonstrates that treatment with recombinant COL17 significantly restored cell viability in a concentration-dependent manner. To elucidate the underlying mechanism, the ROS levels and MMP3 activity were also measured. As illustrated in Figure 5C,D, UVB irradiation at this dosage significantly increased ROS levels and MMP3 activity. Both concentrations of recombinant COL17 (500 and 750 μg/mL) effectively attenuated these UVB-induced elevations in ROS and MMP3.

### 2.6. Protective Role of COL17 Against UV-Induced Epidermal Damage

**Figure 6:**
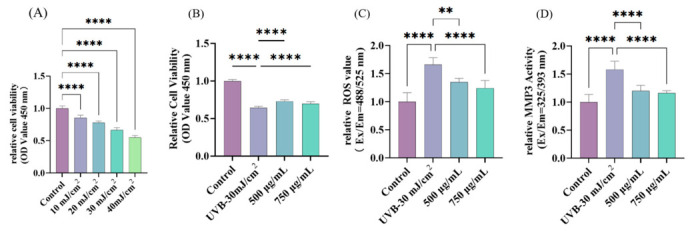
Protective effect of recombinant COL17 on UV-induced epidermal keratinocyte damage. (**A**) Effect of different doses of UVB irradiation on keratinocyte viability. (**B**) Effect of different concentrations of recombinant COL17 on the viability of UVB-irradiated keratinocytes. (**C**) Effect of 30 mJ/cm^2^ UVB irradiation on ROS levels in keratinocytes treated with different concentrations of COL17. (**D**) Effect of 30 mJ/cm^2^ UVB irradiation on MMP3 activity in keratinocytes treated with different concentrations of COL17. Data are presented as mean ± SD (*n* = 3 independent experiments). Statistical significance versus control: ** *p* < 0.01, **** *p* < 0.0001.

Prolonged UV exposure breaks down collagen (a key protein for skin firmness and elasticity) and damages elastin (which enables skin stretching and recoil) [28]. Keratinocytes were cultured in medium containing 500 μg/mL or 750 μg/mL recombinant COL17 and exposed to UVB irradiation to establish a cellular damage model. The levels of ROS and MMP3 were measured to evaluate the protective effect of recombinant COL17 against UVB-induced keratinocyte injury. As shown in Figure 6A, UVB irradiation at doses ranging from 10 to 40 mJ/cm^2^ impaired keratinocyte viability in a dose-dependent manner, and 30 mJ/cm^2^ was selected for subsequent experiments. Figure 6B reveals that treatment with recombinant COL17 at concentrations of 500 and 750 μg/mL effectively restored cell viability impaired by UVB irradiation. The results shown in Figure 6B,C demonstrate that 30 mJ/cm^2^ UVB irradiation significantly increased both intracellular ROS levels and MMP3 activity. Importantly, treatment with recombinant COL17 at both 500 and 750 μg/mL markedly suppressed the UVB-induced elevation of ROS levels and MMP3 activity.

### 2.7. Bioactivity Testing of COL17 via Cell Scratch Assay

**Figure 7:**
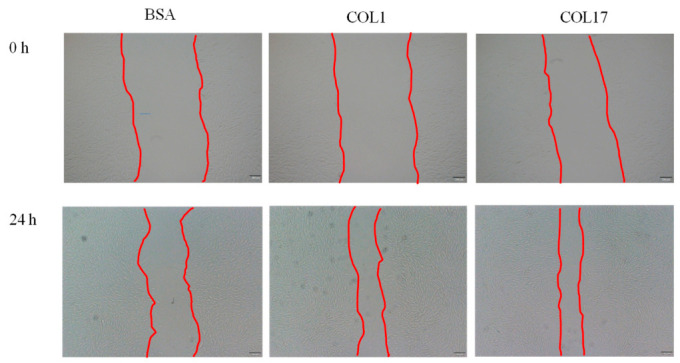
COL17 promotes HFF cell migration in a wound healing assay. Data are presented as mean ± SD (n = 3). Brightness and contrast were adjusted uniformly across all images for clarity. Scale bar = 200 μm. The red line indicates the boundary of the cell scratch.

The cell migration assay was conducted to evaluate the wound healing capacity of HFF cells (Figure 7). After 24 h of incubation, the original wound gap was covered by the migrated cells at 59% and 65% using BSA and COL1 (murine COL1), respectively. In contrast, the COL17 group exhibited a significantly enhanced migration capability. The wound area was markedly reduced, yielding a healing rate of approximately 81%. These results indicate that COL17 effectively promotes HFF cell migration. In UVB-damaged cells, COL17 has been shown to activate TGF-β/Smad while suppressing EGFR/MAPK/AP-1 [13], providing a potential mechanism for the enhanced wound closure observed here.

## 3. Materials and Methods

### 3.1. Strains and Plasmids

The designed collagen fragment is composed of eleven hydrophilic domains (amino acid residues of the α1 chain of COL17). The genes encoding the signal peptide-fused COL17 fragments were codon-optimized, synthesized by General Biosystems, and subsequently cloned into the pPIC9K vector using seamless cloning. Following linearization with Sac I, the recombinant vectors (Table S1) were electroporated into competent *K. phaffii* GS115 cells. Screening for multi-copy transformants was performed on YPD plates supplemented with 2–4 mg/mL G418.

### 3.2. Shake Flask Fermentation

The single colonies of recombinant *K. phaffii* were picked from the agar plates and inoculated into 5 mL of YPD medium (1% yeast extract, 2% peptone, and 2% dextrose), followed by overnight incubation at 30 °C with shaking at 250 rpm to prepare seed cultures. Subsequently, 2 mL of the seed culture was transferred into 50 mL of BMGY medium (1% yeast extract, 2% peptone, 1% glycerol, 400 μg/L biotin, and 0.1 M potassium phosphate, pH 6.0) at 28 °C with shaking at 250 rpm for 24 h. The cells were then centrifuged and resuspended in 50 mL BMMY medium (1% yeast extract, 2% peptone, 400 μg/L biotin, 1% methanol, and 0.1 M potassium phosphate, pH 6.0) to induce COL17 expression for 3 days. To maintain an adequate methanol concentration, 1 mL of 50% (*v*/*v*) methanol was added to the culture medium twice per day. Meanwhile, 0.5 mL of the culture medium was sampled and centrifuged at 10,000 rpm for 5 min at 4 °C. The resulting supernatant was then subjected to SDS-PAGE analysis.

### 3.3. Fed-Batch Fermentation

A stock culture of *K. phaffii* was grown to an OD_600_ of 3–6 in a 500 mL shake flask containing 50 mL YPD. The shake flask culture was used to inoculate a 5 L fermenter containing 2 L of basal salt medium (BSM) with glycerol as the carbon source, supplemented with PTM1 trace elements. The dissolved oxygen level (DO) was set at 25% and the stirring rate ranged from 300 to 800 rpm to balance oxygen demand. The pH of the medium was maintained at 5.8–6.0 through the automatic addition of 25% (*w*/*v*) NH_3_·H_2_O and 5% antifoam as required. Temperature was maintained at 30 °C. The initial cultivation terminated when all glycerol was consumed (about 16 h) at the batch phase. Continuous 50% glycerol feeding was carried out for about 6 h in the subsequent fed-batch phase. Then, methanol induction was initiated with anhydrous methanol containing PTM1 at 6 mL/h, followed by a 24 h adaptation period. The feed rate was then ramped stepwise to 12 mL/h via 48 h post-induction. Culture temperature was reduced to 25 °C during induction to enhance protein stability.

### 3.4. Purification of Recombinant Collagen

The fermentation broth was centrifuged at 8000× *g* and 4 °C for 30 min. The resulting supernatant was subjected to ammonium sulfate precipitation (56.8% *w*/*v*) at 4 °C. After stirring for 30 min, the mixture was centrifuged at 12,000× *g* for 10 min at 4 °C. The pellet was resuspended in deionized water, decolorized with activated charcoal, and filtered through a 0.45 μm membrane. The product was then filtered through an ultrafiltration membrane with a molecular weight cutoff of 3 kDa to remove salt ions. Further purification was performed via size-exclusion chromatography on a Sephadex G-25 column equilibrated with 10 mM Tris-HCl (pH 7.4). Elution was monitored at 245 nm; the main peak fraction was collected, buffer-exchanged into deionized water through another ultrafiltration, sterile-filtered through a 0.22 μm membrane, and lyophilized.

To determine the concentration of the target protein, samples were analyzed via high-performance liquid chromatography (HPLC), and HPLC analysis was performed using a size-exclusion chromatography (SEC-HPLC) method on a Xtimate SEC-300 column (300 mm × 7.8 mm, 5 μm). The mobile phase consisted of phosphate buffer and acetonitrile (10:1, *v*/*v*) at a flow rate of 0.5 mL/min. The column temperature was maintained at 25 °C, and detection was monitored at 220 nm with a 10 μL injection volume. A calibration curve was generated using a purified standard for quantification. The main peak of the target protein eluted at approximately 21.6 min. The concentration of the target protein in the supernatant was calculated based on the peak area of the standard curve.

### 3.5. Cell Lines and Reagents

HFF and HaCaT cells were obtained from the Shanghai Institutes for Biological Sciences (Shanghai, China) and cultured in complete DMEM (Procell, Wuhan, China; Cat# PM150210) with 10% FBS (Procell, Cat# 164210-50) and 1% Penicillin–Streptomycin–Amphotericin B (Servicebio, Wuhan, China; Cat# G4015) at 37 °C with 5% CO_2_. For the CCK-8 assay, cells were seeded into 96-well plates at a density of 2 × 10^3^ cells/well. After 24 h, cells were treated with COL17 at concentrations of 500 ng/mL, 750 ng/mL, 1 μg/mL, 50 μg/mL, 250 μg/mL, 500 μg/mL, 750 μg/mL, and 1 mg/mL. Control cells received the same volume of vehicle (PBS). Cell viability was assessed after treatment for 24, 48, and 72 h. Briefly, 10 μL of CCK-8 reagent was added to each well and incubated for 2 h before measuring the absorbance.

### 3.6. Establishment of a UVB Irradiation-Induced Model of Cell Damage

A UVB injury model was established in HFF and HaCaT cells. Cells were seeded in 96-well plates, cultured for 24 h, and then exposed to graded UVB doses (10–50 mJ/cm^2^). After a further 24 h culture, cell viability was assessed using the CCK-8 assay to determine the optimal irradiation dose for subsequent experiments. To evaluate the protective effect of COL17, the HFF and HaCaT cells were pretreated with various concentrations of COL17 for 24 h, irradiated with the predetermined UVB dose, and then cultured for another 24 h.

### 3.7. Protective Effect of COL17 Against UVB-Induced Dermal Damage

The model was constructed by subjecting cells to UVB (30 mJ/cm^2^) irradiation, as described in 2.5. After UVB irradiation, the cells were changed to a serum-free medium containing different concentrations of recombinant COL17. The unirradiated control group did not receive any treatment, and the UVB-treated group was only treated with UVB irradiation. Following a 48 h incubation period for all experimental groups, the experimental protocol was terminated, and cellular samples were systematically collected for subsequent analysis. All cell experiments were performed three times. The cells were then harvested for the analysis of intracellular ROS levels using a commercial assay kit (Abcam, Shanghai, China) and to determine MMP3 enzymatic activity using a fluorescent activity assay kit (Abcam, Shanghai, China).

### 3.8. RT-qPCR

After 48 h of culture at 37 °C, the total RNA of HFF and HaCaT cells was extracted using the TRIzol reagent and reverse-transcribed into cDNA using PrimeScript™ RT Master Mix (Takara Bio Inc., Kusatsu, Japan). Finally, real-time quantitative PCR (qPCR) was carried out to measure gene expression: in fibroblasts, the expression of key dermal ECM components and remodeling regulators, specifically type I collagen (*COL1A1*), type III collagen (*COL3A1*), and tissue inhibitor of metalloproteinase 1 (*TIMP1*), was assessed; in keratinocytes, the expression of differentiation markers and structural barrier proteins, including keratins (*KRT1*, *KRT5*, *KRT10*, *KRT14*), involucrin (*IVL*), loricrin (*LOR*), and filaggrin (*FLG*), was detected. The primer sequences used in this work are listed in Table S2.

### 3.9. Cell Scratch Assay

HFF cells were seeded in culture plates and cultured until 100% confluence. A linear scratch was created using a sterile pipette tip, and the cells were washed with PBS to remove debris. The medium was then replaced with serum-free medium containing COL17. After incubation for 24 h, images of the wound areas were captured at 0 h and 24 h using an inverted microscope. The wound closure rate was quantified using ImageJ software v1.54r.

### 3.10. Statistical Analysis

Data are presented as the mean ± standard deviation (SD) of three independent experiments. Statistical analyses were performed using GraphPad Prism 10 software. Comparisons between two groups were conducted using the unpaired Student’s *t*-test. For comparisons involving multiple groups, one-way analysis of variance (ANOVA) followed by Tukey’s post hoc test was used. A *p*-value < 0.05 was considered statistically significant.

## 4. Conclusions

By combining codon optimization, signal peptide screening, and multi-copy integration, efficient secretory expression of COL17 was attained in *K. phaffii*, reaching 4.7 g/L in a 5 L fed-batch bioreactor. The recombinant COL17 promoted the proliferation of skin fibroblasts and keratinocytes, enhanced the expression of extracellular matrix (ECM) genes, and upregulated the expression of genes associated with epidermal barrier function. In a UVB-induced photoaging model, it reduced ROS levels and inhibited MMP3 expression, demonstrating significant antioxidant activity. Given its antioxidant capacity and dual functions in facilitating skin repair and supporting barrier-related gene expression, this engineered collagen represents a promising candidate for advanced skincare formulations and biomaterials targeting photoaging protection and skin regeneration.

## Data Availability

The datasets generated and/or analysed during the current study are available from the corresponding authors on reasonable request.

## Supplementary Materials

The following supporting information can be downloaded at: https://www.mdpi.com/article/10.3390/ijms27125613/s1.
