# Supplementary material for: High-Level Secretory Expression of Recombinant Type XVII Human-like Collagen in Komagataella phaffii
Source: Int J Mol Sci. 2026 Jun 22;27(12):5613. doi: 10.3390/ijms27125613 (PMC13299338; doi:10.3390/ijms27125613)
Supplement: Supplementary file 1 [file ijms-27-05613-s001.zip › ijms-4279413-supplementary.pdf]

International Journal of
Molecular Sciences

Table S1. The eleven selected hydrophilic regions of the α1 chain of human COL17.

Amino acid residue sites	Sequence	Grand average of hydropathicity (GRAVY)
575-583	GSPGPKGDR	-1.900
599-613	GPQGPKGQKGSVGDP	-1.447
629-657	GPRGEAGPPGSGEKGERGAAGEPGPHGPP	-1.403
703-717	GPPGPKGDQGEKGPR	-2.080
829-849	GPPGPPGAPGPAGPAGLPGHQ	-0.624
905-919	GPPGPPGPPGPKGDQ	-1.607
950-973	GPPGPPGPQGPKGDKGDPGVPGAL	-0.886
1218-1235	GPPGPPGPRGPPGVSGAL	-0.506
1265-1279	GPPGPPGPQGPPGDS	-1.400
1399-1410	GPPGQPGPQGPP	-1.517
1438-1482	GPPGQKGEMGTPGPKGDRGPAGPPGHPGP<br>P<br>GPRGHKGEKGDKGDQ	-1.778

Table S2. Primer sequences used in this study.

Primer Name	Forward Sequence (5'→3')	Reverse Sequence (3'→5')
KRT1	ATCAACTACCAGCGCAGGAC	GTTGGTCCACTCTCCTTCGG
KRT5	GTCTTGCCGGAGGTAGCAG	AAGAGGCAATCTCCATGGGC
KRT10	GACAAAGTTCGGGCTCTGGA	CCCCTGATGTGAGTTGCCAT
KRT14	CCAGTTCTCCTCTGGATCGC	TCCAGTGGGATCTGTGTCCA
IVL-1	CAGGCCAGGTCCAAGACATT	GGATTGGGGTCATTGGGGTT
LOR1	GTGAGCTCAGACTTGGTGCT	CAAGGGCCCAGATGTCCAAT
FLG	ATCTGAGGGCACTGAAAGGC	CACTTCCGTGCTGAGAGTGT
COL1A1	TCCCAGGGTGGCTTCTGATA	GAGTCGGGGACACTTACAGC
COL3A1	CACGTCTACATTAAGGAACTCCA	GTCCAGTATGTCCCACAGGG
TIMP1	TTGGCTGTGAGGAATGCACA	GTCCACAAGCAATGAGTGCC

Table S3. Information on the α-MF secretion signals used in this study.

Species	Abbreviation	α-factor pre-pro peptides	Sequence
Saccharomyces cerevisiae	*S. ce*	MRFPSIFTAVLFAASSALAAPVNTTED<br>ETAQIPAEAVIGYSDLEGDFDVAVLPFS<br>NSTNNGLLFINTTIASIAAKEEGVSLEK<br>R	ATGAGATTTCCTTCAATTTTTACTGCAGTTTTATTCGCAGCATC<br>CTCCGCATTAGCTGCTCCAGTCAACACTACAACAGAAGATGA<br>AACGGCACAAATTCCGGCTGAAGCTGTCATCGGTTACTCAGA<br>TTTAGAAGGGGATTTCGATGTTGCTGTTTTGCCATTTTCCAAC<br>AGCACAAATAACGGGTTATTGTTTATAAATACTACTATTGCCA<br>GCATTGCTGCTAAAGAAGAAGGGGTATCTCTCGAGAAAAGA
Geotrichum candidum	*G. ca*	MRFSLATVYAFTVIGTVLGVPIASSEPT<br>ATTLSTVAAASATFSPGGDSPFTGIKNF<br>PDFASFPPFPPGFDTGLSKR	ATGAGGTTTTCATTGGCCACTGTATATGCTTTCACTGTAATTGG<br>AACAGTTTTAGGAGTGCCTATTGCTAGTTCTGAGCCAACTGC<br>TACCACTTTGTCTACTGTAGCTGCAGCTTCAGCCACTTTCTCA<br>CCAGGAGGTGACTCTCCATTCACTGGCATCAAGAATTTTCCC<br>GATTTCGCTTCCTTTCCACCCTTTCCACCAGGATTCGATACCG<br>GATTGTCTAAACGT
Lachancea dasiensis	*L. da*	MFREFSAYLTLALAMSRAWAAPVNLS<br>SSLSMNEGQSLPAESIVGFLDLTGADD<br>LALFPVSNATNTGVLIVNTTVLDSATE<br>SKKRGKR	ATGTTCAGAGAGTTTTCAGCTTACCTTACCCTTGCTTTGGCTA<br>TGTCCAGAGCTTGGGCTGCCCCTGTTAACCTGTCCTCCTCAC<br>TTTCAATGAACGAGGGTCAATCTCTGCCTGCCGAATCTATCGT<br>AGGTTTTCTGGATTTGACCGGAGCTGACGATTTGGCCTTGTTC<br>CCTGTTTCTAACGCAACCAATACAGGTGTGCTTATTGTTAACA<br>CGACTGTTCTGGACTCTGCTACTGAATCAAAGAAAAGAGGA<br>AAGCGT
Millerozyma farinosa	*M. fa*	MKFSAIVISGLLGAGLVKAAPVDSGA<br>KGKYSRTDLIIPDEAIANRYVVGDDEQ<br>PVFAEIDNKPVVYIVNTTKAESIVAKSG<br>ITLDDLKESYANATKEEEAKNGKR	ATGAAATTCTCTGCTATTGTCATCTCTGGTCTTTTGGGTGCCG<br>GACTGGTGAAGGCCGCACCCGTAGACTCAGGAGCTAAGGGA<br>AAATACTCTAGAACTGACTTGATTATCCCAGATGAGGCAATCG<br>CTAACAGGTATGTGGTGGGAGATGACGAGCAACCTGTCTTCG<br>CTGAGATTGACAACAAACCAGTGGTTTACATTGTCAACACAA<br>CGAAGGCTGAGTCCATTGTGGCCAAGAGTGGAATTACACTTG<br>ACGATCTTAAGGAGTCTTATGCAAACGCCACCAAGGAGGAA<br>GAAGCAAAGAACGGTAAAAGA
Tetrapisispora phaffii	*T. ph*	MKLSSVLSTLALTATSAFAAPVSNESV<br>DNASVPAEAIIGYLNFDGANDIALLPFS	ATGAAGCTGTCTTCTGTGCTTAGTACCCTGGCCCTTACTGCTA<br>CATCTGCTTTCGCCGCCCCAGTTAGTAATGAGTCTGTTGATAA

		NSTTSGVMFINTTIAEQAYEEAGVSLSKR	CGCATCTGTACCTGCCGAGGCTATAATCGGCTATCTGAACTTCGATGGTGCTAATGACATCGCATTGTTGCCTTTCTCAAACTCTACCACTTCAGGAGTTATGTTCATCAATACGACCATTGCTGAGCAAGCTTACGAGGAGGCAGGTGTCTCCCTATCCAAAAGA
Wickerhamomyces ciferrii	*W. ci*	MQLSLLTSLAIVSTLLGSSFAAPVENINIKDNGNGTSEADVPGTSQGVEFPFAKEAIIEAVSLGNDIAPIVLNDAVYFVNTTTVDKELESKLGKR	ATGCAGCTTTCTCTGCTTACTTCTCTGGCTATTGTTTCAACACTTCTGGGAAGTTCTTTTGCTGCTCCAGTCGAGAACATTAACATCAAGGATAACGGTAACGGAACCTCAGAAGCTGATGTTCCAGGTACTTCTCAGGGTGTGGAGTTCCCCTTTGCCAAGGAAGCAATCATTGAGGCTGTTTCCCTGGGTAACGACATCGCCCCCATTGTTTTGAACGATGCTGTCTATTTCGTTAACACAACTACTGTTGATAAGGAACTTGAGAGTAAGCTTGGTAAACGA
